# A jumping shape memory alloy under heat

**DOI:** 10.1038/srep21754

**Published:** 2016-02-16

**Authors:** Shuiyuan Yang, Toshihiro Omori, Cuiping Wang, Yong Liu, Makoto Nagasako, Jingjing Ruan, Ryosuke Kainuma, Kiyohito Ishida, Xingjun Liu

**Affiliations:** 1 Department of Materials Science and Engineering, College of Materials, Xiamen University, Xiamen, 361005, P.R. China; 2 Department of Materials Science, Graduate School of Engineering, Tohoku University, 6-6-02 Aoba-yama, Sendai 980-8579, Japan; 3 School of Mechanical and Aerospace Engineering, Nanyang Technological University, Singapore 639798; 4 Institute for Materials Research, Tohoku University, 2-1-1 Katahira, Sendai 980-8577, Japan; 5 Collaborative Innovation Center of Chemistry for Energy Materials, Xiamen University, Xiamen 361005, P. R. China

## Abstract

Shape memory alloys are typical temperature-sensitive metallic functional materials due to superelasticity and shape recovery characteristics. The conventional shape memory effect involves the formation and deformation of thermally induced martensite and its reverse transformation. The shape recovery process usually takes place over a temperature range, showing relatively low temperature-sensitivity. Here we report novel Cu-Al-Fe-Mn shape memory alloys. Their stress-strain and shape recovery behaviors are clearly different from the conventional shape memory alloys. In this study, although the Cu-12.2Al-4.3Fe-6.6Mn and Cu-12.9Al-3.8Fe-5.6Mn alloys possess predominantly L2_1_ parent before deformation, the 2H martensite stress-induced from L2_1_ parent could be retained after unloading. Furthermore, their shape recovery response is extremely temperature-sensitive, in which a giant residual strain of about 9% recovers instantly and completely during heating. At the same time, the phenomenon of the jumping of the sample occurs. It is originated from the instantaneous completion of the reverse transformation of the stabilized 2H martensite. This novel Cu-Al-Fe-Mn shape memory alloys have great potentials as new temperature-sensitive functional materials.

Metallic materials when heated are often accompanied with the changes of microstructure, shape or performance, which can be considered as functional materials for various practical applications. Typically, shape memory alloy (SMA) is a kind of temperature-sensitive metallic functional materials, possessing shape memory effect (SME) and superelasticity (SE) characteristics, such as Ni-Ti and Cu-Al-Mn alloys^1^,^2^,^3^,^4^,^5^. The SME and SE behaviors depend on the reversible martensitic transformation, having some differences between them. The SME phenomenon is that the deformation strain in the martensite sample could be recovered through reverse transformation via heating. The SE property is associated with the stress-induced martensitic transformation in the austenite sample and the corresponding immediate reverse transformation upon unloading. Typically, a large stress-plateau upon loading could be observed for above two situations, owing to the reorientation of martensite variants and/or de-twinning for SME^1^,^6^,^7^, or the stress-induced martensitic transformation process for SE^1^,^6^,^8^,^9^, respectively. Furthermore, the shape recovery process and the actuation speed reported in the existing SMAs usually takes place over a temperature range^7^,^10^,^11^,^12^,^13^, showing relatively low temperature-sensitivity.

The transformation temperatures of the existing SMAs that are used in practice are generally not higher than 100 °C, which cannot satisfy the requirements of high temperature applications^10^. To date, several types of high-temperature shape memory alloys (HTSMAs) for high temperature applications have been explored, such as Ni-Ti-Pd/Hf/Zr^14^,^15^,^16^,^17^, Ni-Mn-Ga-based^7^,^11^,^18^, Ni_3_Ta^12^, Zr-Cu-based^13^. Although the polycrystalline Ni-Ti-Pd^14^,^15^ and single crystal Ni-Mn-Ga^7^ alloys exhibit about 5.4% and 6.1% of high temperature shape recovery strains, the high-costs for the precious metal of Pd and for the production of single crystal of Ni-Mn-Ga have hindered their practical applications. Since the raw materials of Cu-Al-based SMAs are much cheaper, they have also attracted much attentions as high-temperature actuation purposes, such as Cu-Al-Nb^19^,^20^, Cu-Al-Ag^21^, Cu-Al-Fe^22^, Cu-Al-Ta^23^, Cu-Al-Co and Cu-Al-Zr^24^ alloys. However, the major drawbacks of these materials are their poor recovery strain and poor thermal stability, which are not desirable when being used at high temperature. These situations motivate us to further explore potential low-cost SMAs that can stably operate at high temperature. Here we present a novel Cu-12.2Al-4.3Fe-6.6Mn (mass%) SMA. It has a giant and stable full shape recovery strain of about 9% when cycling through high temperature (above 100 °C). It is further discovered that the shape recovery process can complete instantly accompanied with the jumping of the sample. Such extraordinary property has never been reported in other types of SMAs.

## Results

### Microstructure and martensitic transformation

**Figure 1:**
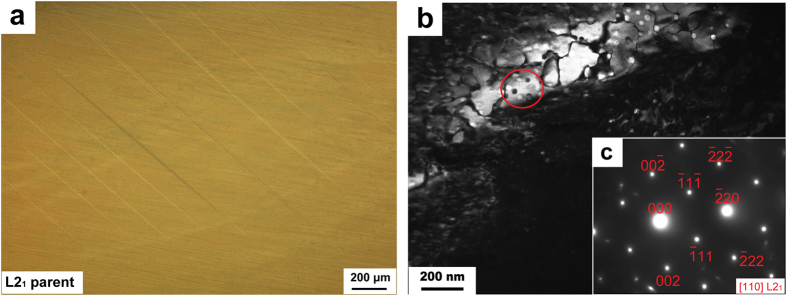
Microstructure of Cu-12.2Al-4.3Fe-6.6Mn alloy. (**a**) Optical micrograph. (**b**) Dark-field image and SADP (**c**) of the parent.

**Figure 2:**
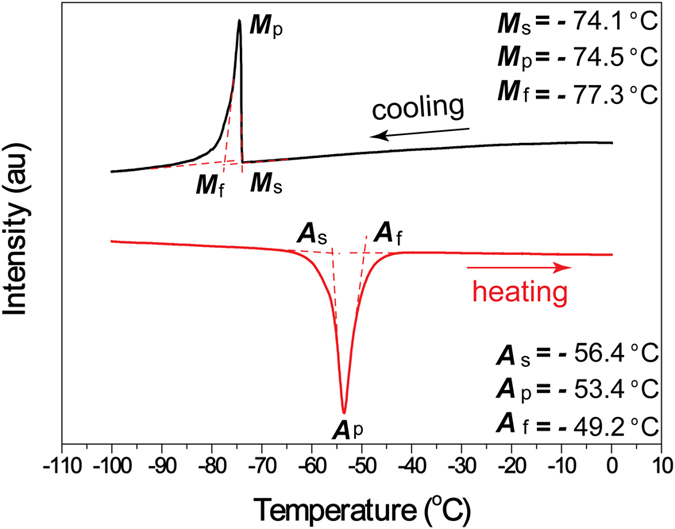
DSC curve of Cu-12.2Al-4.3Fe-6.6Mn alloy. *M*_s_, *M*_p_ and *M*_f_ are the martensite transformation starting, peak and finishing temperatures, *A*_s_, *A*_p_ and *A*_f_ are the austenite transformation starting, peak and finishing temperatures, respectively. au, arbitrary units.

Figure 1 shows the microstructure of Cu-12.2Al-4.3Fe-6.6Mn alloy (see the Methods). This sample consists of dominate parent (Fig. 1a) and a small amount of needle-like martensite (Figure S1a). The transmission electron micrograph (TEM) and the selected area diffraction pattern (SADP) of the parent shown in Fig. 1b,c. The parent is confirmed to be an ordered L2_1_ structure, which is similar to those observed in Cu-Al-Mn SMAs^4^,^5^,^25^. The martensite has a 2H (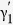) structure based on the TEM image and SADP in Figure S1b, c. Additionally, in Fig. 1b, several fine precipitation particles are observed as indicated by a red circle. These particles are further identified to be two typed precipitates of A2 and ordered D0_3_ structures^25^,^26^,^27^ (see the Figures S2 and S3 for the details). The martensitic transformation temperatures of the sample were determined by DSC test as shown in Fig. 2. The temperatures *M*_s_, *M*_p_ and *M*_f_, *A*_s_, *A*_p_ and *A*_f_ are −74.1 °C, −74.5 °C and −77.3 °C, −56.4 °C, −53.4 °C and −49.2 °C, respectively. The transformation thermal hysteresis *T*_h_ (*T*_h_ = *A*_f_ − *M*_s_) is only 24.9 °C.

### Stress-strain behaviors

**Figure 3:**
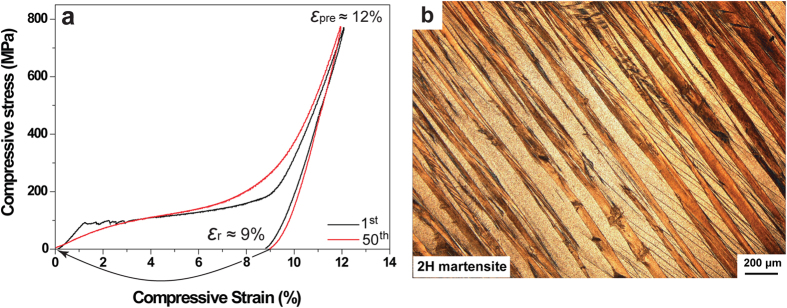
(**a**) Compressive stress-strain curves of Cu-12.2Al-4.3Fe-6.6Mn alloy during the 1^st^ (black) and the 50^th^ (red) thermomechanical cycles. The arrow represents the recovery strain of the deformed sample by heating. (**b**) Microstructure of Cu-12.2Al-4.3Fe-6.6Mn alloy after compressed to a pre-strain of 12% and unloading.

Figure 3a shows the compressive stress-strain curves of Cu-12.2Al-4.3Fe-6.6Mn alloy with a pre-strain of 12% during the 1^st^ (black) and the 50^th^ (red) thermomechanical cycles. Figure 3b exhibits the microstructure of the deformed sample after compressed to a pre-strain of about 12% and unloading. It is noted that the sample almost consists of martensite at the same time. The martensite has 2H (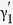) structure by electron diffraction (Figure S4a, b). Here, a novel stress-strain behavior is found in Cu-12.2Al-4.3Fe-6.6Mn alloy. The usual SE property associates with the stress-induced martensitic transformation^1^,^6^,^8^,^9^, though the transformation temperatures are significantly below testing temperature. Meanwhile, the reverse transformation immediately occurs during unloading process, along with the shape recovery. Interestingly, we find although the studied sample possesses predominant L2_1_ parent before deformation, the stress-induced strain (namely the large stress-plateau during loading in Fig. 3a) does not recover during unloading accordingly. The stress-induced 2H martensite from L2_1_ parent could be retained after unloading. Such martensite could be called the “stabilized stress-induced martensite”.

**Figure 4:**
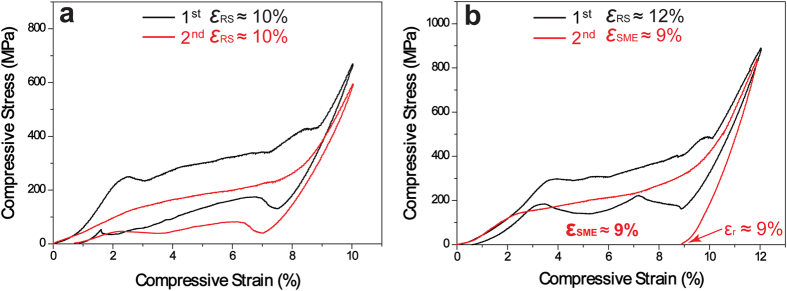
Compressive stress-strain curves of Cu-12.9Al-3.8Fe-5.6Mn alloy with two cycles under different pre-strains of 10% (**a**) and 12% (**b**).

**Figure 5:**
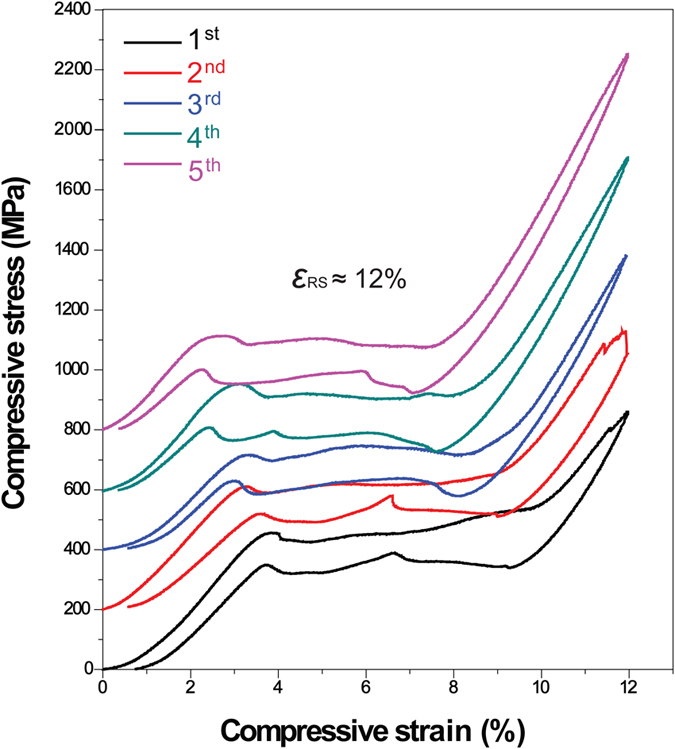
Compressive stress-strain curves of Cu-13.5Al-2.9Fe-6.8Mn alloy with five cycles under the same pre-strain of 12%.

With the decrease of the transformation temperature by changing the alloy compositions, other Cu-Al-Fe-Mn alloys exhibit clearly different stress-strain behaviors. Figure S5 shows the reversible martensitic transformation temperatures of Cu-12.9Al-3.8Fe-5.6Mn alloy, which are lower than those of Cu-12.2Al-4.3Fe-6.6Mn alloy in Fig. 2. Two samples were prepared and different compressive pre-strains of 10% and 12% were performed with two cycles, and the results are provided in Fig. 4. When a 10% of compressive pre-strain is performed during two cycles, full shape recovery strain (*ε*_RS_) of 10% is obtained after the removal of load in Fig. 4a. Increasing the compressive pre-strain to 12% in Fig. 4b, the sample still shows full shape recovery strain (*ε*_RS_) of 12% during the first test indicated by the black line. However, during the second cycle, the “stabilized stress-induced martensite” process occurs, in which the residual strain is also about 9% indicated by the red line. With further decreasing the transformation temperature, the normal SE behavior is observed in Cu-13.5Al-2.9Fe-6.8Mn alloy (The martensitic transformation temperatures of Cu-13.5Al-2.9Fe-6.8Mn alloy are much lower than above two alloys, in which there are no transformation peaks cannot be detected by DSC test between −150 °C and 20 °C). Figure 5 is the compressive stress-strain curves of Cu-13.5Al-2.9Fe-6.8Mn alloy with five cycles. The same compressive pre-strain of 12% was performed before each cycle in the same sample. Here, the stress-induced strain during loading immediately recovers after the removal of load. Compared to other two studied alloys, the difference is that the “stabilized stress-induced martensite” process does not take place, whereas it suggests an excellent shape recovery strain (*ε*_RS_) of about 12%.

### Shape recovery characteristics

**Figure 6:**
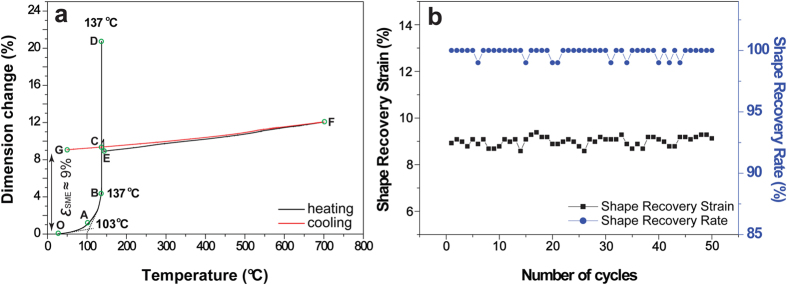
Shape recovery properties of Cu-12.2Al-4.3Fe-6.6Mn alloy. (**a**) TMA curve of the deformed sample with the residual strain of about 9% during the 1^st^ cycle. (**c**) Shape recovery strains and rates of the deformed sample during fifty thermomechanical cycles.

The shape recovery characteristics of Cu-12.2Al-4.3Fe-6.6Mn sample were investigated through thermomechanical cycles. The same compressive pre-strain (ε_pre_) of 12% was performed before each cycle (Fig. 3a). Then the deformed sample was heated to introduce reverse transformation and the dimensional change was recorded by a thermal mechanical analyser (TMA). Figure 6a shows the shape recovery curve of the sample during the 1^st^ thermal cycle. It is observed that the initial shape recovery starts within stage A-B (around 103–137 °C). However, the remaining residual strain recovers completely and instantly at 137 °C (stage B-C-D). At this stage, the sample jumps strongly during shape recovery, see the Video S1. Subsequently, fifty thermomechanical cycles were performed between room temperature and 700 °C in air. As shown in Fig. 6b, nearly perfect shape recovery strain of about 9% and shape recovery rate of about 100% are obtained each time, indicating excellent thermomechanical stability. The shape recovery behavior of the deformed specimen of Cu-12.9Al-3.8Fe-5.6Mn in Fig. 4b is similar to that of Cu-12.2Al-4.3Fe-6.6Mn alloy, see the Video S2. In Videos S1 and S2, the deformed sample is heated on a heating platform exposed to the air; the temperature at which the “jumping” of the samples occurs at 152 °C and 55 °C for Cu-12.2Al-4.3Fe-6.6Mn and Cu-12.9Al-3.8Fe-5.6Mn alloys, respectively. Such shape recovery properties have never been reported in other types of SMAs, in which the deformation strain of the present alloy can recover instantly and completely, showing extreme “temperature-sensitivity”.

## Discussion

**Figure 7:**
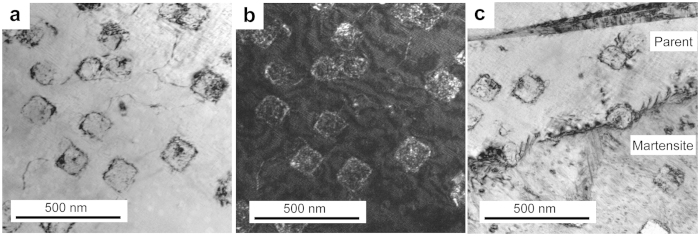
TEM images of Cu-12.2Al-4.3Fe-6.6Mn alloy after compression and heating up to 140 °C. (**a**) Bright-field image in parent phase. (**b**) Weak-beam dark-field image in parent phase. (**c**) Bright-field image in parent and martensite phases.

The novel stress-strain characteristics of the present quaternary Cu-Al-Fe-Mn SMAs are clearly different from the usual SE property. In this study, we think that the movement of the habit plane causing reverse transformation is probably inhibited by very fine precipitates, and/or the dislocations formed during deformation. Figure S6 shows the transmission electron microscopy-energy dispersive spectrometer (TEM-EDS) mapping analysis of the compressed Cu-12.2Al-4.3Fe-6.6Mn specimen and heated up to 140 °C. It is clearly seen that those Fe-rich particles exhibit the grain size of 10–30 nm in spherical shape (similar to those in Figure S2) and 100–200 nm in cuboidal shape respectively. Furthermore, the microstructure of the specimen is observed using TEM tests. Figure 7 shows the bright-field image (a) and weak-beam dark-field image (b) of the parent phase, the bright-field image in parent and martensite phases (c). From Fig. 7 it is found that a band of dislocation is observed close by those precipitates with the size of about 100 nm. Thus although the testing temperature is far above their *A*_f_ temperatures in Fig. 3, the introduced dislocations probably stabilize the martensite after the removal of the load. As we known, the decrease of Fe content should reduce the volume fraction of the Fe-rich precipitates in the BCC phase separation^26^. So the amounts of Fe-rich precipitates decrease. This should result in the decrease of the martensitic transformation temperatures because that more Fe and Mn are retained in the parent^4^,^5^. As a result, the resistance to the movement of habit plane during reverse transformation reduces, and it results in a “normal” SE property of the alloys in Figs 4a and 5. Furthermore, with the increases of deformation level and mechanical cycling times, the more dislocations should be formed, thus the resistance to the movement of habit plane increases accordingly. It may also induce the phenomenon of the “stabilized stress-induced martensite” in Fig. 4b.

In our view, the shape recovery behaviors of Cu-12.2Al-4.3Fe-6.6Mn and Cu-12.9Al-3.8Fe-5.6Mn alloys are also different from the conventional SME. The above studied two alloys exhibit extreme “temperature-sensitivity”, in which the deformation strain after unloading can recover instantly and completely accompanied with the jumping of the sample. We consider the mechanism of jumping behavior based on the TEM images as follows. The precipitates play as obstacles for movement of habit plane during reverse transformation. The stress-induced martensite during loading is stabilized by the precipitates and the introduced dislocations. Therefore, the barrier for the habit plane movement becomes higher. The reverse transformation starts by building up the driving force from the inevitable trend of reverse martensitic transformation during heating. When a critical temperature is reached, the driving force is large enough to overcome the resistance of the pinners. In this situation, the suppressed reverse martensitic transformation can proceed instantly and completely with a full shape recovery. At the same time, the phenomenon of the “jumping” of the samples happens.

## Conclusions

In summary, the presently developed low-cost Cu-12.2Al-4.3Fe-6.6Mn and Cu-12.9Al-3.8Fe-5.6Mn SMAs possess unique stress-strain and shape recovery characteristics. The large recovery strain of about 9% is originated from the reverse transformation of the stabilized stress-induced 2H martensite triggered through heating. The shape recovery process is extremely temperature-sensitive upon heating and can complete instantly when a critical temperature is reached. Our findings suggest that the present alloys can be considered to be used as new metallic functional materials, such as high sensitivity detector or sensor. Especially, Cu-12.2Al-4.3Fe-6.6Mn alloy exhibits a recovery temperature higher than 100 °C and excellent thermomechanical stability, which also offers great promise for high temperature applications.

## Methods

Three samples with the chemical compositions of Cu-12.2Al-4.3Fe-6.6Mn, Cu-13.5Al-2.9Fe-6.8Mn and Cu-12.9Al-3.8Fe-5.6Mn (mass%) alloys were prepared by arc melting under an argon atmosphere using a non-consumable tungsten electrode. The ingots were cut into small bulks and sealed into vacuum quartz ampoules, and annealed at 900 °C for 24 h followed by quenching into ice-water. The chemical composition analysis was determined by electron probe microanalysis (EPMA) and transmission electron microscopy-energy dispersive spectrometer (TEM-EDS). The microstructure was observed by optical microscopy (OM) and transmission electron micrographs (TEM). The crystal structure was identified by TEM and the selected area diffraction pattern (SADP). The martensitic transformation temperatures were determined by differential scanning calorimetry (DSC) at a heating and cooling rate of 10 °C min^−1^.

Cylindrical specimens (3 mm diameter, 5 mm height) were cut from the quenched bulks. The shape recovery properties were performed by compressive tests. The thermomechanical stability of Cu-12.2Al-4.3Fe-6.6Mn alloy was measured by thermomechanical cycles. Before each cycle, the same sample was compressed to a pre-strain of 12% at room temperature and unloading, then followed by heating to about 700 °C for 2 min and cooling back to room temperature for one cycle. The shape recovery during the 1^st^ cycle was measured by thermal mechanical analysis (TMA) under an argon atmosphere at a heating and cooling rate of 10 °C min^−1^. The subsequent cycles for the same sample were taken up in air until to fifty times. The height of the sample was measured before loading (*h*_0_), after unloading (*h*_1_) and after recovery (*h*_2_). The shape recovery strain and rate were calculated as: (*h*_2_ − *h*_1_)/*h*_0_ × 100% and (*h*_2_ − *h*_1_)/(*h*_0_ − *h*_1_) × 100%, respectively.

## Additional Information

**How to cite this article**: Yang, S. *et al.* A jumping shape memory alloy under heat. *Sci. Rep.* **6**, 21754; doi: 10.1038/srep21754 (2016).

## Supplementary Material

Supplementary video 1

Supplementary video 2

Supplementary Information
