# Supplementary material for: A jumping shape memory alloy under heat
Source: Sci Rep. 2016 Feb 16;6:21754. doi: 10.1038/srep21754 (PMC4754943; doi:10.1038/srep21754)
Supplement: Supplementary Information [file srep21754-s3.pdf]

Supplementary Information

A jumping shape memory alloy under heat

Shuiyuan Yang[1,]*, Toshihiro Omori[2], Cuiping Wang[1], Yong Liu[3], Makoto Nagasako[4], Jingjing Ruan[1], Ryosuke Kainuma[2], Kiyohito Ishida[2] & Xingjun Liu[1,5,]*

[1] Department of Materials Science and Engineering, College of Materials, Xiamen University, Xiamen, 361005, P.R. China

[2] Department of Materials Science, Graduate School of Engineering, Tohoku University, 6-6-02 Aoba-yama, Sendai 980-8579, Japan

[3] School of Mechanical and Aerospace Engineering, Nanyang Technological University, Singapore 639798

[4] Institute for Materials Research, Tohoku University, 2-1-1 Katahira, Sendai 980-8577, Japan

[5] Collaborative Innovation Center of Chemistry for Energy Materials, Xiamen University, Xiamen 361005, P. R. China

* Corresponding authors: yangshuiyuan@xmu.edu.cn(S.Y. Yang); lxj@xmu.edu.cn (X.J. Liu)

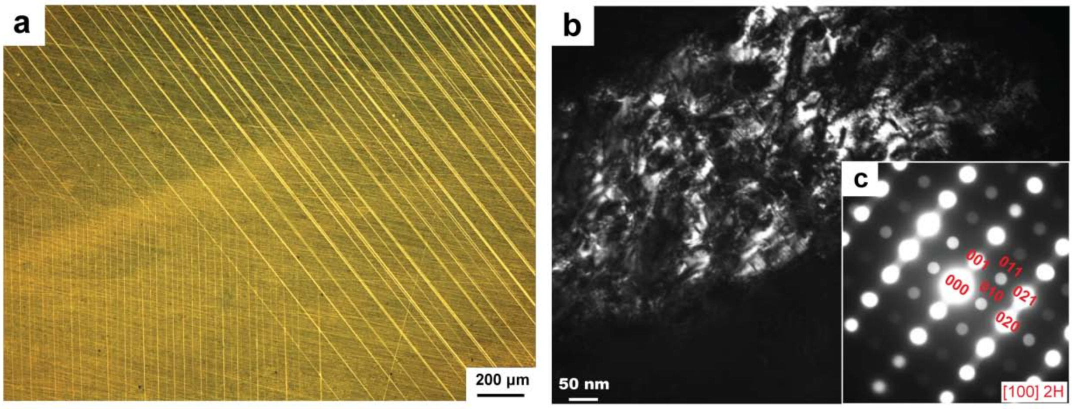

Figure S1. Optical micrograph (a), dark-field image (b) and SADP (c) of the martensite in Cu-12.2Al-4.3Fe-6.6Mn alloy. The results show that Cu-12.2Al-4.3Fe-6.6Mn alloy after quenching contains a small amount of needle-like martensite. The martensite is identified to have a 2H structure based on TEM image and SADP in (b) and (c).

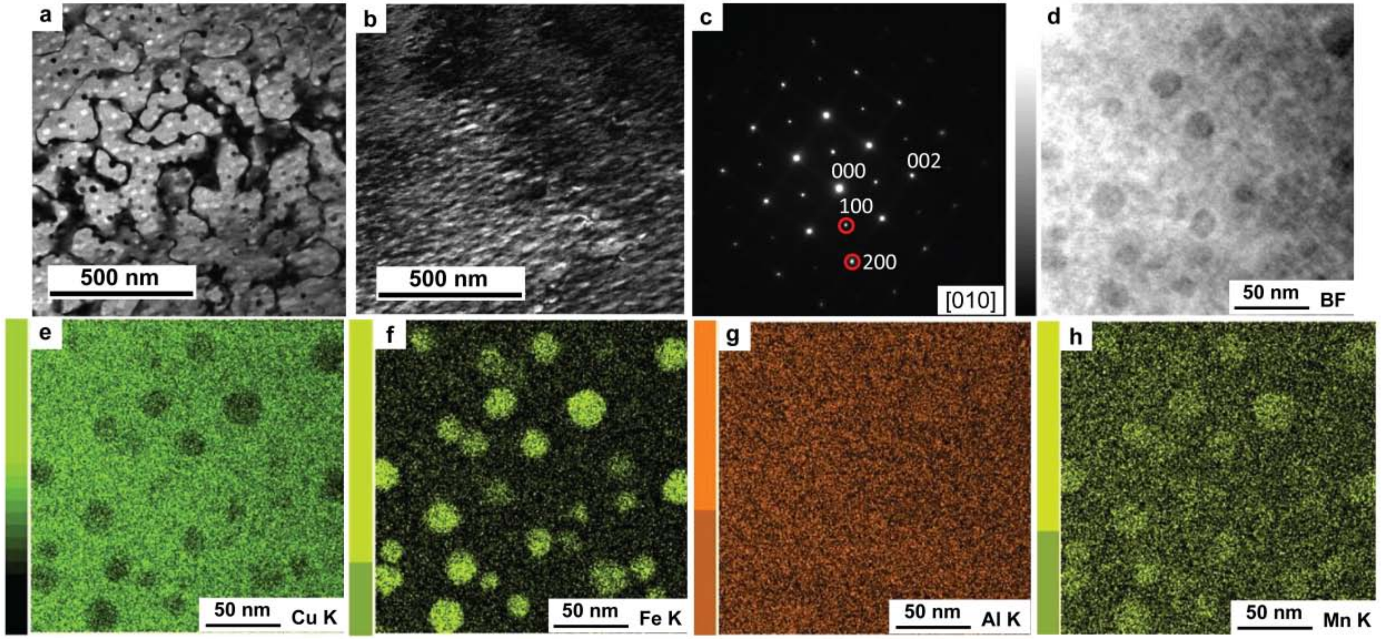

Figure S2. Electron micrographs (a-c) and transmission electron microscopy-energy dispersive spectrometer (TEM-EDS) mapping analysis (d-h) of the precipitates in Cu-12.2Al-4.3Fe-6.6Mn alloy after quenching. (a-c) Dark-field image obtained using the superlattice reflection 100 (a), fundamental reflection 200 (b) and its SADP (c) of the particles; (d) - (h) TEM-EDS mapping analysis of the particles. The results show that the matrix ($L2_1$, Cu_2MnAl) and the precipitation particles are respectively Cu-rich and Fe-rich, while Al content is almost the same in the matrix and the precipitates. This result agrees well with the reported phase diagram of Cu-Fe-Al system showing the BCC phase separation[26]. It is noted that the Mn is more enriched in the precipitates. It is considered that the precipitates were formed in the $L2_1$ (Cu_2MnAl) matrix during cooling from 900 °C. It is believed that the grain size and distribution of these precipitation particles are dependent on the cooling condition. One can clearly see the two types of particles with bright and dark contrast in the dark-field image (a), but no dark particles were observed in the dark-field image obtained using fundamental reflection 200 (b). From (a) - (c), it is found that one of two typed particles is the A2 precipitate. In addition, we took the HAADF-STEM image of the particle and the result confirmed the existence of another ordered $D0_3$ precipitate in Figure S3. Therefore, the Cu-12.2Al-4.3Fe-6.6Mn alloy has the A2 and the ordered $D0_3$ precipitates. The two types of precipitates are explained by the phase separation of the BCC phase into A2 + $D0_3$ in Fe-Mn-Al system[27].

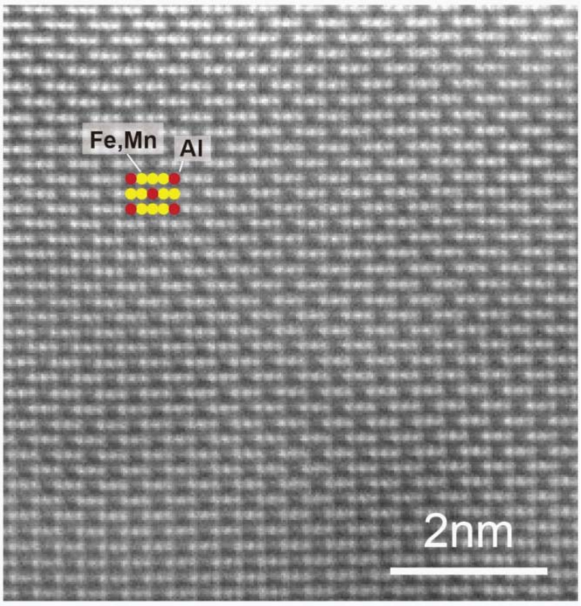

Figure S3. HAADF-STEM image of the precipitates in Cu-12.2Al-4.3Fe-6.6Mn alloy after compression and heating up to 200 °C, incident beam direction being [110]. Because the intensity of the atomic column is almost proportional to the square of the average atomic number *Z*, it is found that the atomic columns with dark contrast are occupied by Al and that those with bright contrast are by Fe and Mn. Therefore, the crystal structure of the particle can be determined to be ordered $D0_3$ structure.

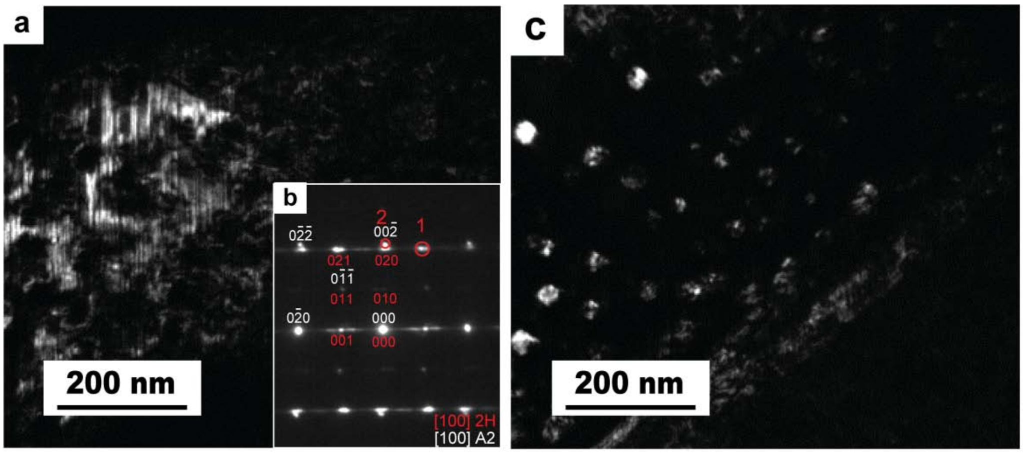

Figure S4. Dark-field image (a) and SADP (b) of the martensite when Cu-12.2Al-4.3Fe-6.6Mn alloy is compressed to a pre-strain of about 12% and unloading. It is confirmed that the sample mainly possesses a 2H martensite structure at the same time. Additionally, fine precipitation particles are also observed in (c).

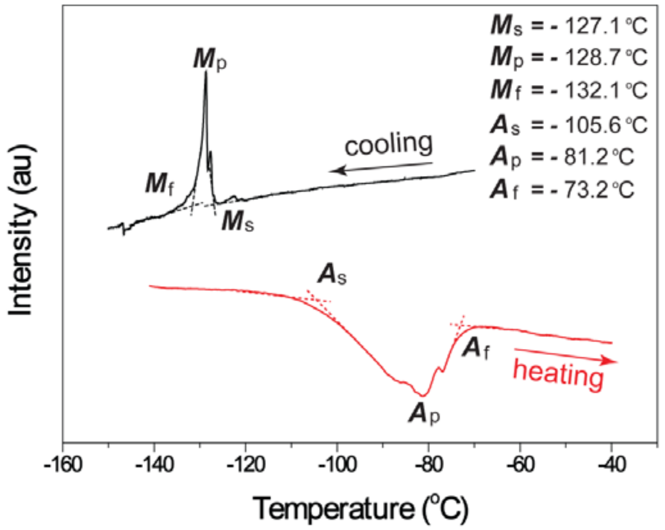

Figure S5. DSC curve of Cu-12.9Al-3.8Fe-5.6Mn alloy. The reversible martensitic transformation temperatures are lower than those of Cu-12.2Al-4.3Fe-6.6Mn alloy (Figure 2). M_s, M_p and M_f are the martensite transformation starting, peak and finishing temperatures, A_s, A_p and A_f are the austenite transformation starting, peak and finishing temperatures, respectively. au, arbitrary units.

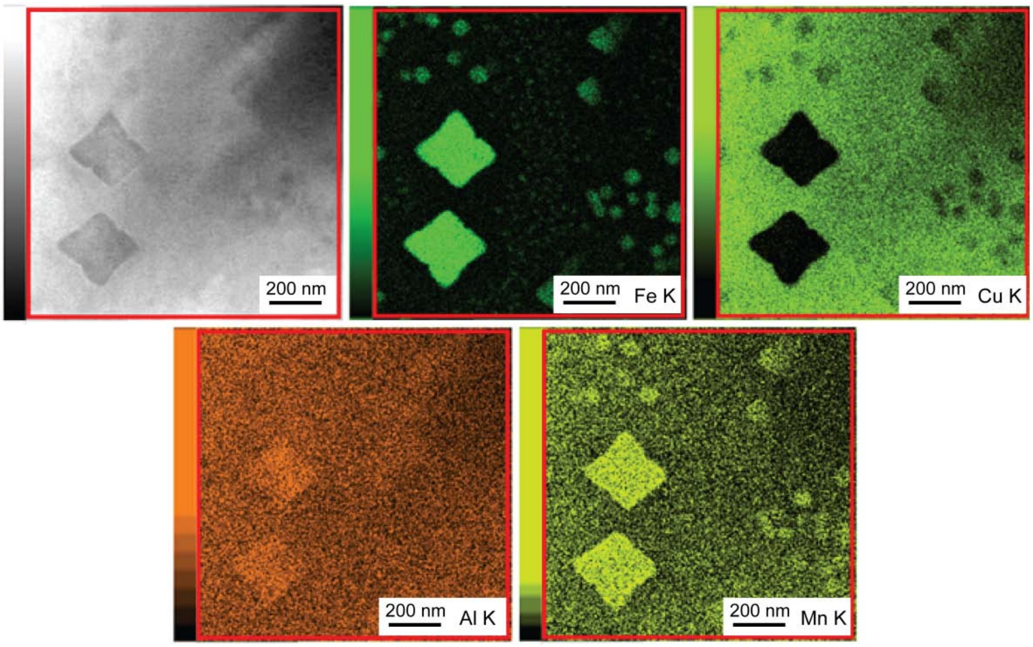

Figure S6. Transmission electron microscopy-energy dispersive spectrometer (TEM-EDS) mapping analysis of Cu-12.2Al-4.3Fe-6.6Mn alloy after compression and heating up to 140 °C.

Video S1. Shape recovery behavior of Cu-12.2Al-4.3Fe-6.6Mn alloy.

This video records the shape recovery behavior of a Cu-12.2Al-4.3Fe-6.6Mn specimen (3 mm diameter, 5 mm height) after compressed to a pre-strain (ε_{pre}) of 12% at room temperature and unloading. The residual strain (ε_r) is about 9%. The deformed sample is heated on a heating platform exposed to the air. The temperature at which the “jumping” of the samples occurs at 152 °C, and the residual strain recovers completely and instantly ($\varepsilon_{SME} \approx 9\%$).

Video S2. Shape recovery behavior of Cu-12.9Al-3.8Fe-5.6Mn alloy.

This video records the shape recovery behavior of a Cu-12.9Al-3.8Fe-5.6Mn specimen (3 mm diameter, 5 mm height) after compressed to a pre-strain (ε_{pre}) of 12% with two cycles at room temperature (see Fig. S2c). During the first cycle, this alloy shows a full shape recovery strain ($\varepsilon_{RS} \approx 12\%$) after unloading. However, after the second cycle, the residual strain (ε_r) is about 9% after unloading. The deformed sample is heated on a heating platform exposed to the air. The temperature at which the “jumping” of the samples occurs at 55 °C, and the residual strain recovers completely and instantly ($\varepsilon_{SME} \approx 9\%$).
